# Comprehensive Screening of Eight Known Causative Genes in Congenital Hypothyroidism With Gland-in-Situ

**DOI:** 10.1210/jc.2016-1879

**Published:** 2016-08-15

**Authors:** Adeline K. Nicholas, Eva G. Serra, Hakan Cangul, Saif Alyaarubi, Irfan Ullah, Erik Schoenmakers, Asma Deeb, Abdelhadi M. Habeb, Mohammad Almaghamsi, Catherine Peters, Nisha Nathwani, Zehra Aycan, Halil Saglam, Ece Bober, Mehul Dattani, Savitha Shenoy, Philip G. Murray, Amir Babiker, Ruben Willemsen, Ajay Thankamony, Greta Lyons, Rachael Irwin, Raja Padidela, Kavitha Tharian, Justin H. Davies, Vijith Puthi, Soo-Mi Park, Ahmed F. Massoud, John W. Gregory, Assunta Albanese, Evelien Pease-Gevers, Howard Martin, Kim Brugger, Eamonn R. Maher, V. Krishna K. Chatterjee, Carl A. Anderson, Nadia Schoenmakers

**Affiliations:** University of Cambridge Metabolic Research Laboratories (A.K.N., E.S., G.L., V.K.K.C., N.S.), Wellcome Trust-Medical Research Council Institute of Metabolic Science, Addenbrooke's Hospital, Cambridge, United Kingdom; Department of Human Genetics (E.G.S., C.A.A.), The Wellcome Trust Sanger Institute, Hinxton, Cambridge, United Kingdom; Research Centre for Regenerative and Restorative Medicine (H.C.), Department of Medical Genetics Istanbul Medipol University, Kavacık, Istanbul, Turkey; Pediatric Endocrine Unit (S.A., I.U.), Department of Child Health, Sultan Qaboos University Hospital, Muscat, Oman; Paediatric Endocrinology Department (A.D.), Mafraq Hospital, AbuDhabi, United Arab Emirates; Pediatric Department Prince Mohamed Bin Abdulaziz Hospital (A.M.H.), Madinah, Kingdom of Saudi Arabia; Department of Paediatrics (M.A.), Madina Maternity & Children's Hospital Madina Munawara, Saudi Arabia; 8. Department of Endocrinology (C.P.), Great Ormond St Hospital for Children, London, United Kingdom; Department of Paediatrics (N.N.), Luton and Dunstable University Hospital, Luton, United Kingdom; Division of Paediatric Endocrinology (Z.A.), Dr Sami Ulus Woman Health and Children Research Hospital Ankara, Turkey; Department of Paediatric Endocrinology (H.S.), Uludağ University, School of Medicine Bursa, Turkey; Department of Paediatric Endocrinology (E.B.), Dokuz Eylül University, Faculty of Medicine Izmir, Turkey; Developmental Endocrinology Research Group (M.D.), Section of Genetics and Epigenetics in Health and Disease, Genetics and Genomic Medicine Programme, University College London Institute of Child Health, London, United Kingdom; Department of Paediatrics (S.S.), Leicester Royal infirmary, Leicester United Kingdom; Centre for Paediatrics and Child Health (P.G.M.), Institute of Human Development University of Manchester, and Royal Manchester Children's Hospital, Manchester, United Kingdom; Paediatric Endocrinology Division (A.B.), College of Medicine, King Saud University and King Saud University Medical City, Riyadh, Saudi Arabia; Department of Paediatrics (R.W., A.T.), University of Cambridge, Cambridge Biomedical Campus, Cambridge, United Kingdom; W Midlands Regional Genetics Laboratory (R.I.), Birmingham Women's Hospital NHS Foundation Trust, Birmingham, United Kingdom; Department of Paediatric Endocrinology (R.P.), Central Manchester University Hospitals NHS Foundation Trust, Manchester, United Kingdom; Department of Paediatrics (K.T.), Diana Princess of Wales Hospital, Grimsby, United Kingdom; Department of Paediatric Endocrinology (J.H.D.), University Hospital Southampton, Southampton, United Kingdom; Department of Paediatrics (V.P.), Peterborough and Stamford Hospitals NHS Foundation Trust, Peterborough, United Kingdom; Department of Clinical Genetics (S.-M.P.), Cambridge University Hospitals NHS Foundation Trust, Cambridge United Kingdom; London N W Healthcare NHS Trust (A.F.M.), Harrow, Middlesex, United Kingdom; Division of Population Medicine (J.W.G.), School of Medicine, Cardiff University, Heath Park Cardiff, UK; Department of Paediatric Endocrinology (A.A.), St George's University Hospitals NHS Foundation Trust, London, United Kingdom; Centre for Endocrinology (E.P.-G.), William Harvey Research Institute, Queen Mary University London and Children's Hospital, Barts Health NHS Trust, London, United Kingdom; Department of Medical Genetics (H.M., K.B., E.R.M.), University of Cambridge and NIHR Cambridge Biomedical Research Centre, Cambridge, United Kingdom

## Abstract

**Context:**

Lower TSH screening cutoffs have doubled the ascertainment of congenital hypothyroidism (CH), particularly cases with a eutopically located gland-in-situ (GIS). Although mutations in known dyshormonogenesis genes or *TSHR* underlie some cases of CH with GIS, systematic screening of these eight genes has not previously been undertaken.

**Objective:**

Our objective was to evaluate the contribution and molecular spectrum of mutations in eight known causative genes (*TG, TPO, DUOX2, DUOXA2, SLC5A5, SLC26A4*, *IYD,* and *TSHR*) in CH cases with GIS.

**Patients, Design, and Setting:**

We screened 49 CH cases with GIS from 34 ethnically diverse families, using next-generation sequencing. Pathogenicity of novel mutations was assessed in silico.

**Results:**

Twenty-nine cases harbored likely disease-causing mutations. Monogenic defects (19 cases) most commonly involved *TG* (12), *TPO* (four), *DUOX2* (two), and *TSHR* (one). Ten cases harbored triallelic (digenic) mutations: *TG* and *TPO* (one); *SLC26A4* and *TPO* (three), and *DUOX2* and *TG* (six cases). Novel variants overall included 15 *TG*, six *TPO*, and three *DUOX2* mutations. Genetic basis was not ascertained in 20 patients, including 14 familial cases.

**Conclusions:**

The etiology of CH with GIS remains elusive, with only 59% attributable to mutations in *TSHR* or known dyshormonogenesis-associated genes in a cohort enriched for familial cases. Biallelic *TG* or *TPO* mutations most commonly underlie severe CH. Triallelic defects are frequent, mandating future segregation studies in larger kindreds to assess their contribution to variable phenotype. A high proportion (∼41%) of unsolved or ambiguous cases suggests novel genetic etiologies that remain to be elucidated.

## Abstract (precis)

TG, TPO, DUOX2, DUOXA2, SLC5A5, SLC26A4, IYD and TSHR genes were screened in 49 cases of congenital hypothyroidism with eutopic gland-in-situ. 59% were solved including cases with triallelic mutations.

Congenital hypothyroidism (CH) is the most common neonatal endocrine disorder, and, historically, thyroid dysgenesis was thought to account for approximately 80% of cases (1). However, recent studies have reported a change in the epidemiology of CH, with a doubling in incidence to around 1 in 1500 live newborns, predominantly driven by an increase in CH with eutopic gland-in-situ (GIS), which accounted for almost two-thirds of recently diagnosed cases in Lombardy, Italy (2). Lower TSH screening cutoffs may be the major driver for this increase in diagnosis, although altered ethnicities of the screened population, increased multiple and premature births, iodine status, and hitherto uncharacterized factors may also contribute (3, 4).

The molecular basis of CH with GIS remains poorly understood (5, 6). Genetic variation in seven genes involved in thyroid hormone biosynthesis (*TG, TPO, DUOX2, DUOXA2, IYD, SLC5A5,* and *SLC26A4*) and *TSHR* mediates some cases. Disease-causing mutations are usually biallelic, with the exception of monoallelic *DUOX2*, *IYD,* and *TSHR* mutations, which may also confer a phenotype (1). Phenotypic heterogeneity in cases harboring similar causative mutations suggests that mono- and polygenic factors and environmental modulators may also play a role in determining disease severity (7, 8).

Genetic characterization of CH with GIS has been limited by the cost and labor implications of Sanger sequencing multiple exons. Previous studies have generally focused on either a small number of genes (eg, *TG, TPO, TSHR,* and *DUOX2* in 43 Korean cases) (6), specific phenotypic subsets of cases (5, 8), or multiple genes in a small subset of patients (9). There are occasional reports of digenic mutations involving *TSHR* and either *DUOX2* (6, 10, 11) or *TPO* (12), or combined *DUOX2* and *DUOXA2* mutations (13). However, the role of oligogenicity in disease development and penetrance remains unclear, with no evidence for an additive effect of digenic mutations in one large published kindred (12).

Next-generation sequencing (NGS) technologies increase sequencing capacity and speed, enabling efficient screening of multiple genes simultaneously. A recent publication describes large-scale multiplexed genetic screening of *TPO, TSHR, DUOX2, DUOXA2, PAX8,* and *SLC5A5* in 170 Korean CH cases. However, cases were from a single ethnic background and not selected on the basis of thyroid morphology; moreover *TG, IYD,* and *SLC26A4* were not sequenced (11). We undertook comprehensive screening of *TG, TPO, DUOX2, DUOXA2, IYD, SLC5A5*, *SLC26A4,* and *TSHR* in an ethnically and biochemically heterogeneous CH cohort with GIS. In addition to reporting known and novel mutations in these genes, we document the frequent occurrence of potential oligogenicity, with triallelic variation in two candidate genes, in a population enriched for familial and consanguineous cases.

## Patients and Methods

### Patients

All investigations were part of an ethically approved protocol and/or clinically indicated, being undertaken with written informed consent from patients and/or next of kin including specific consent for whole exome sequencing (WES) (MREC 98/5/024). Forty-nine cases were included in the study from 34 families referred from centers in the United Kingdom, Oman, Saudi Arabia, the United Arab Emirates, and Turkey. Inclusion required clinical evidence of goiter or radiological evidence of a normally sited thyroid gland in the proband. In five cases without goiter who had not undergone thyroid imaging at diagnosis, we accepted goiter or radiological evidence of GIS in at least one affected family member with CH, assuming a common underlying genetic etiology. A diagnosis of overt or subclinical primary CH was made on the basis of referral through newborn screening and/or a raised venous TSH. Newborn screening blood spot cutoffs were as follows: 6–10 mU/liter (United Kingdom), 10 mU/liter (United Arab Emirates), or cord blood TSH 40 mU/liter (Oman). Childhood TSH normal range was 0.35–5.5 mU/liter. Thyroid biochemistry was measured using local analyzers in the referring hospitals.

### DNA sequencing

Three different NGS-based strategies (whole-exome sequencing, WES, and two different targeted sequencing protocols) were used to screen *TG, TPO*, *TSHR, DUOX2*, *DUOXA2*, *IYD*, NIS (*SLC5A5*), and pendrin *SLC26A4*. Detailed methods, coverage, and quality control data are available in the Supplemental Methods and Results. We sought to identify rare variants (minor allele frequency < 0.02 in all control databases) with likely pathogenic consequences predicted by in silico algorithms. Given the ethnic heterogeneity of our cohort, we selected the maximum number of control exomes (n = ∼80, 000) matched as closely for ethnicity as we could achieve (Supplemental Methods). All positive results were validated by Sanger sequencing.

### Nomenclature

Variants were described using nomenclature approved by the Human Genome Variation Society (http://www.HGVS.org/varnomen). Further details are available in the Supplemental Methods.

### Structural model for TPO and DUOX2

The models for TPO and DUOX2 were generated using the phyre2 (Protein Homology/analogy Recognition Engine 2) web portal, which predicts and analyses protein structures based on homology/analogy recognition to solved protein crystal structures (14). The figures were generated with MacPyMOL Molecular Graphics System, Schrödinger, LLC.

## Results

### Sequencing data quality

Detailed information regarding individual gene coverage is summarized in the (Supplemental Results). In the samples sequenced by WES or HiSeq targeted sequencing panel, optimal median coverage (>30 fold) was achieved for all genes except *DUOXA2* and *SLC5A5* in the eleven samples screened by targeted sequencing (median coverage 5-fold and 24-fold respectively) (Supplemental Figure 1A, B). Exons screened using the MiSeq targeted sequencing panel either achieved a more than 20-fold coverage (in house validation had demonstrated 100% sensitivity for detecting variants at this sequencing depth), or were repeated by Sanger sequencing, such that this approach was expected to be highly sensitive. In the WES and HiSeq protocols, in common with previous studies employing similar techniques, although median coverage was generally acceptable, coverage was nonuniform across individual genes (Supplemental Figure 2). This was most marked with the HiSeq targeted sequencing panel in which specific exons exhibited a less than 10-fold coverage, including *DUOXA2* (exons 1, 2, 4, 5, and 6), *SLC5A*5 (exons 1–3, 5, 6, 11, 12, and 15), *DUOX2* (exons 2, 5, 6, 8, 15, and 34), *TG* (exons 13, 15, and 16), *TPO* (exons 3, 7, 8, and 16), *SLC26A4* (exon 21), and *IYD* (exon 6). A detailed comparison of the sequencing techniques is provided in Supplemental Figure 2.

### Mutation frequencies (Figure 1)

**Figure 1.**
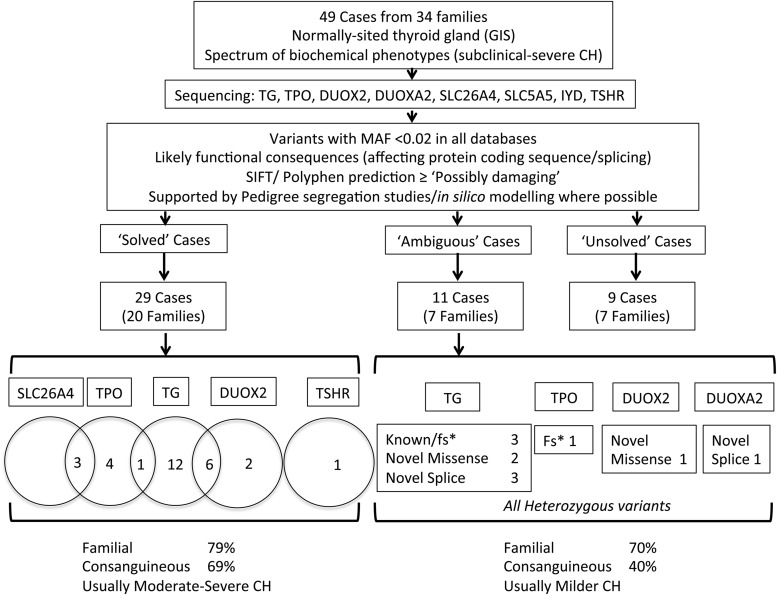
Schematic illustrating case selection, variant filtering, and distribution of mutations in the cohort of patients studied with CH and GIS. “Solved” cases refers to cases in whom a definitive link was established between genotype and CH phenotype. In “ambiguous” cases, the ascertained genotype could plausibly be contributing to the phenotype, but the evidence to support a causal link was weaker than in the “solved” group, and “unsolved” cases carried no mutations in any of the listed genes. The numbers of cases harboring monoallelic or biallelic mutations in each gene are listed beneath the corresponding gene name for the “solved” cases. Numbers in the intersect between circles denote triallelic cases harboring mutations in both genes. In the “ambiguous” category, the number of mutations in each gene is classified by mutation type beneath the relevant gene name; all except DUOXA2 were monoallelic. “Solved” and “ambiguous” or “unsolved” cases were equally likely to be familial, but CH was generally more severe in the “solved” cases. fs*; frameshift mutation resulting in a premature stop codon; MAF, minor allele frequency; splice; splice region variant, VUS, variant of uncertain significance.

Forty-nine cases from 34 families of European, Asian, Middle Eastern, and Afro-Caribbean origin were investigated and 29 cases (20 families, 59%) were considered “solved” following identification of a decisive link between genotype and phenotype. In 11 “ambiguous” cases (22%), it was felt that the ascertained genotype could plausibly be contributing to the phenotype, but the evidence to support a causal link was weaker than in the “solved” group. Finally, nine cases were considered “unsolved” because they carried no mutations in any of the listed genes. Detailed genetic and phenotype data are supplied in Supplemental Tables 1, 2, and 3.

CH was more severe biochemically in solved cases than in unsolved or ambiguous cases (mean TSH, 100 mU/liter vs 36 mU/liter at diagnosis, *P* = .02, Welch's *t* test) and solved cases were more frequently from consanguineous backgrounds (69% cases vs 40% cases). This likely reflects the increased incidence of recessive disease in the presence of consanguinity because CH-associated mutations in five of the eight targeted genes (*TG, TPO, DUOXA2, SLC5A5,* and *SLC26A4*) are usually biallelic. Cases with affected siblings were common in both solved and unsolved or ambiguous categories (79% vs 70% cases) (Figure 1, Supplemental Tables 2 and 3).

### “Solved” kindreds harboring mutations in one gene (monogenic kindreds)

Nineteen cases had a monogenic basis of disease, most commonly involving biallelic mutations in *TG* (12 cases), followed by *TPO* (four cases), *DUOX2* (one monoallelic and one biallelic mutation), and *TSHR* (one case). There were no cases with CH attributable to mutations in *IYD, SLC5A5,* or *SLC26A4* (Figure 1).

### TG mutations (Figure 2)

**Figure 2.**
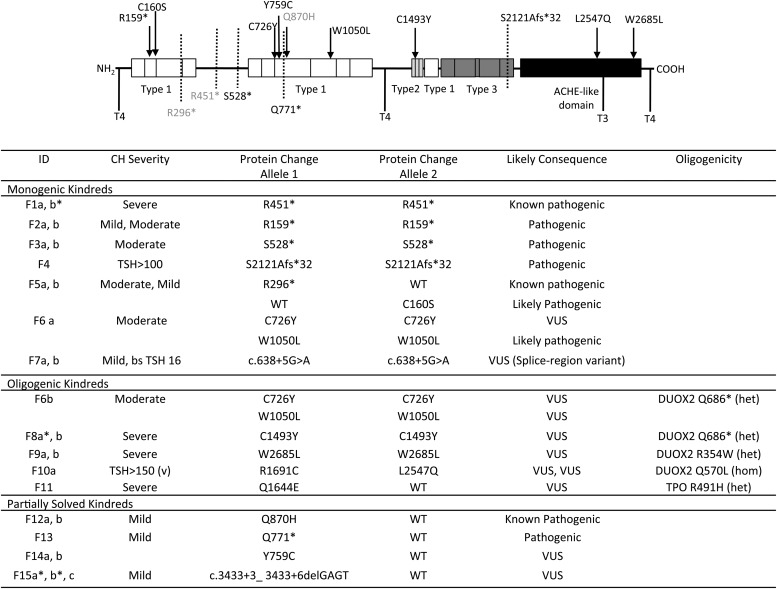
Summary of *TG* mutations identified in the study and the associated biochemical phenotype. CH severity is classified according to European Society for Paediatric Endocrinology criteria on the basis of serum fT4 levels; severe, <5, moderate 5 to <10, and mild >10 pmol/liter, respectively (33) and pathogenicity is predicted according to American College of Medical Genetics guidelines (34). A schematic of the TG protein illustrates the position of the mutations relative to the key structural domains of TG including the repetitive type 1, 2, and 3 cysteine-rich regions, acetylcholinesterase homology (ACHE-like) domain and hormonogenic domains. Known mutations are shown in gray, novel mutations in black. *Cases for which complete biochemical data at diagnosis is not available. CH severity refers to sibling. bs, blood spot.

TG is the secretory protein upon which thyroid hormone is synthesized, and the 12 cases with monogenic *TG* mutations predominantly exhibited moderate-severe CH (Figure 2). One known and three novel homozygous nonsense or frameshift mutations were identified which truncate TG before the carboxy-terminal acetyl cholinesterase (ACHE)-like domain, which has a crucial role in normal conformational maturation and intracellular trafficking of TG (F1, 2, 3, 4) (15). Two siblings (F5a, b) were compound heterozygous for a known nonsense mutation (p.R296*) and a rare, novel missense variant, (p.C160S) that affects a highly conserved cysteine residue in TG (Genomic Evolutionary Rate Profiling score 5.84). Cysteine residues within repetitive domains in the TG form intramolecular disulphide bonds needed for protein folding; thus, p.C160S may be deleterious to TG affecting the tertiary structure as predicted by PolyPhen (16–18). Two siblings (F7a, b) harbored the same homozygous *TG* splice region variant (c.638+5 G>A) inherited from heterozygous parents; although the pathogenicity of this cannot be ascertained *in silico*, it is unique to the affected siblings, and adjacent to a known pathogenic mutation (c.638+1G>A) (19), supporting causality, albeit in association with a mild CH phenotype.

### TPO mutations (Figure 3)

**Figure 3.**
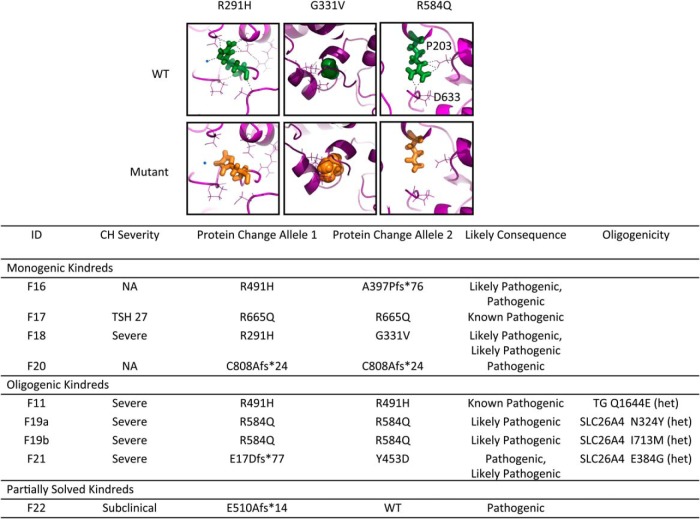
Summary of *TPO* mutations identified in the study and the associated biochemical phenotype. CH severity is classified according to European Society for Paediatric Endocrinology criteria (33) and pathogenicity is predicted according to American College of Medical Genetics guidelines (34). The effect of the novel missense mutations was modeled using the phyre2-server. Figures in the top row show the wild-type (WT) model, with amino acids of interest in green; figures on bottom row show the model with the mutant amino acid (orange); local polar contacts are shown with black broken lines. The R291H and R584Q mutations affect amino acids contributing to an intensive network of H-bond contacts close to the catalytic domain involving the heme-group. R291 makes polar contacts with R585 and R582, interacting directly with the heme-group and R584 makes direct polar contacts with the heme-group itself as well as P203 and D633. The mutations R291H (increased hydrophobicity) and R584Q (resulting in a smaller polar group) are likely to disrupt polar contacts affecting local structure and are predicted to affect catalytic activity. The G331V mutation affects local space filling with the larger valine predicted to impair substrate binding by displacement of the nearby helix and/or disruption of polar contacts (orange amino acids, H_2_O molecules in blue), affecting the local structure of TPO.

TPO is the heme peroxidase catalyzing the final steps of thyroid hormone synthesis, and biallelic mutations (Figure 3) were identified in four monogenic kindreds. These included two known pathogenic missense mutations (F16; p.R491H, F17; p.R665Q), two novel frame shift (F20; p.C808Afs*24, F16; p.A397Pfs*76), and two novel missense variants (F18; p.R291H, p.G331V) (Table 2). The p.R291H variant is predicted to disrupt a hydrogen bond network close to the TPO heme group thereby destabilizing the catalytic domain. G331 is located close to the substrate binding domain, and mutation to the larger valine amino acid will likely cause steric hindrance impeding substrate binding (Figure 3). Two cases were compound heterozygous: F16 p.[A397Pfs*76];[R491H], associated with dyshormonogenic goiter requiring thyroidectomy, and F18 p.[R291H];[G331V], who also exhibited goiter.

### DUOX2 mutations (Figure 4)

**Figure 4.**
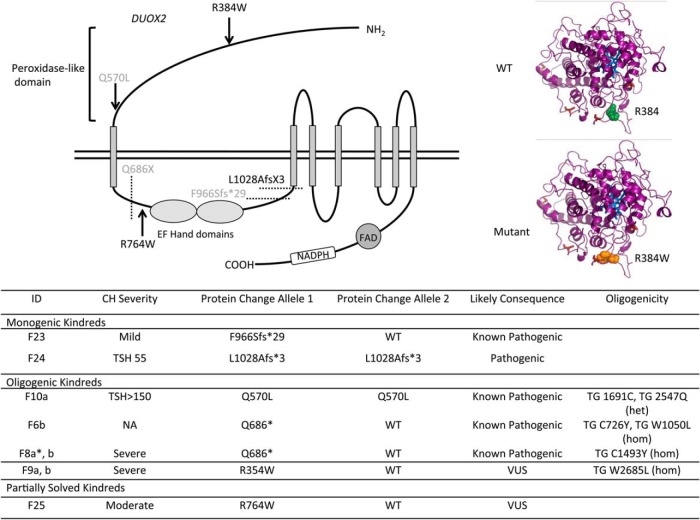
Summary of *DUOX2* mutations identified in the study and the associated biochemical phenotype. CH severity is classified according to European Society for Paediatric Endocrinology criteria (33) and pathogenicity is predicted according to American College of Medical Genetics guidelines (34). Mutation position is illustrated using a schematic representation of the domain structure of the DUOX2 protein. Known mutations are shown in gray and novel mutations in black. The structural model of the peroxidase domain suggests that R354 is part of an intensive hydrogen network. The novel missense mutation R354W replaces the hydrophilic arginine by the hydrophobic tryptophan disrupting this network and also results in a possible repositioning of the loop containing R354 and C351, which mediates interactions between the peroxidase domain and extracellular loops obligatory for DUOX2 function.

DUOX2 is the nicotinamide adenine dinucleotide phosphate oxidase, which generates H_2_O_2_ required for thyroid hormone biosynthesis. Two solved cases with monogenic *DUOX2* mutations were identified (Figure 4), including one known heterozygous mutation (F23; p.F966Sfs*29) and one novel homozygous mutation (F24; p.L1028Afs*3), both of which would truncate DUOX2 before the nicotinamide adenine dinucleotide phosphate oxidase domain, thereby abrogating protein function. Affected cases generally had a milder or transient (F23) CH phenotype compared with cases harboring monogenic *TG* and *TPO* mutations.

### TSHR mutation

A single individual from the United Arab Emirates with mild CH harbored a known pathogenic heterozygous *TSHR* mutation (F26; p.P68S) (Supplemental Table 2), previously identified in an Arab population. Parental DNA was not available; however, the mild CH phenotype was consistent with previously reported biochemistry associated with this mutation (20).

### “Solved” kindreds harboring mutations in two genes (oligogenic kindreds, Figure 5)

**Figure 5.**
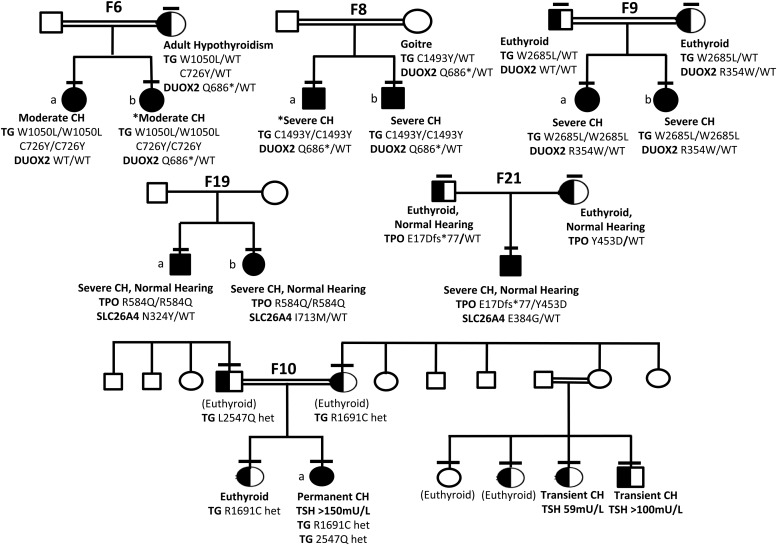
Genotype-phenotype segregation in six kindreds with oligogenic variants. Horizontal bars denote individuals who have been genotyped. Black shading denotes homozygous individuals and half-black shading denotes heterozygotes for *TG* mutations (F9, F6, F8), TPO mutations (F19, F21), and *DUOX*2 mutations (F10). Potential oligogenic modulators are included by aligning genotype and phenotype data with the individual to whom they refer in the pedigree. *Cases for whom complete biochemical data at diagnosis are not available (F6b, F8a); CH severity refers to sibling. In F10, black, half-black, and white shading denote the *DUOX2* genotype (Q570L homozygous, heterozygous, or wild-type, respectively). The pedigree is annotated with *TG* genotype in those cases harboring variants (L2547Q, R1691C), and phenotype (euthyroid, transient, or permanent CH) with venous screening TSH results for CH cases. Cases annotated (euthyroid) were born in Pakistan and although euthyroid in adulthood; that they were not screened neonatally for CH may have precluded detection of transient CH.

Ten solved cases from seven families harbored digenic pathogenic variants. These were predominantly triallelic, and most commonly comprised biallelic *TG* mutations in association with a monoallelic *DUOX2* mutation. Such digenic mutations were detected in consanguineous Turkish kindreds F6, 8, and 9 (Figure 5). In these kindreds, although defined as variants of uncertain significance by ACMG criteria, the biallelic *TG* mutations were rare (p. W1051L; MAF <0.001 in 1KG Europeans, and absent in all other population datasets, including ExAC East Asians) or unique, affected conserved amino acids and were predicted to be pathogenic by PolyPhen and SIFT. In F6, two siblings (a, b) with CH were both homozygous for *TG* p.W1051L and p.C726Y but one sibling (F6b) harbored an additional, maternally inherited heterozygous *DUOX2* mutation (p.Q686*), previously described in association with transient CH (21). Biochemistry at diagnosis could not be retrieved from F6b for comparison with F6a; however, both presented with neonatal goiter and had similar levothyroxine requirements. Their mother exhibited adult-onset hypothyroidism of unknown etiology. Two unrelated sibling pairs also harbored homozygous *TG* mutations in association with a heterozygous *DUOX2* mutation: *TG* p.1493Y and *DUOX2* p.Q686* in F8a, b and *TG* p.W2685L and *DUOX2* R354W (predicted to perturb the DUOX2 peroxidase-like domain) in F9a, b (Figure 4). There was also a strong history of goiter (mother and maternal aunt) in F8 but maternal DNA was not available to confirm *DUOX2* genotype. In all three kindreds, the most severe phenotype was observed in individuals harboring biallelic *TG* or triallelic (biallelic *TG* and monoallelic *DUOX2*) mutations; however, it was impossible to distinguish the effects of the mutations in the two genes reliably in these small pedigrees with limited subphenotype data.

Since monogenic, heterozygous *DUOX2* mutations (including p.Q686*) are frequently associated with CH, we hypothesized that an additive phenotypic contribution of all three mutations was very plausible. Calculation of the number of East Asian individuals in the ExAC database (n = 8654) harboring similarly rare, predicted damaging variants in *DUOX2* yielded a population mutation frequency of 0.06%. The observed proportion of *TG* mutation carriers with a monoallelic *DUOX2* variant in our cohort (8.8% families) was therefore significantly higher (*P* = .0233, Fisher's exact one-tailed test), supporting a potential phenotypic contribution of the *DUOX2* mutation in these individuals. Much larger cohorts of sequenced CH individuals will be required to assess the phenotypic consequences of digenicity in CH thoroughly.

Biallelic mutations in *TPO* were identified in two kindreds in addition to heterozygous known *SLC26A4* mutations, previously associated with recessive disease: F19a: *TPO* p.R584Q (homozygous) and *SLC26A4* p.N324Y (heterozygous); F19b: *TPO* p.R584Q (homozygous) and *SLC26A4* p.I713M (heterozygous); F21: *TPO* p.[E17Dfs*77]; [Y453D] (compound heterozygous); and *SLC26A4* p.E384G (heterozygous) (Figure 5). The novel *TPO* p.R584Q missense variant is predicted to perturb polar contacts possibly affecting the catalytic domain (Figure 4).

The occurrence of Pendred syndrome usually mandates biallelic *SLC26A4* mutations, and manifests universally with congenital or postnatal progressive sensorineural hearing loss, whereas thyroid dysfunction is usually mild or absent. In both these kindreds, only the biallelic *TPO* mutations segregated with CH; this was severe whereas hearing was normal. In F11, a known homozygous pathogenic *TPO* mutation (p.R491H) was inherited together with a heterozygous *TG* variant (p.Q1644E). Because biallelic inheritance is also usually required for CH due to *TG* mutations, these observations suggest the *TPO* mutations are predominant drivers of the CH phenotype in these three kindreds, although we cannot definitively exclude a contribution of the heterozygous *SLC26A4* or *TG* mutation. Comparison with population mutation frequencies in *TG* and *SLC26A4* in the ExAC cohort (non-Finnish Europeans, N = 66,740), suggested that congruence of *TPO* mutations with *TG* or *SLC26A4* mutations was not increased in our cohort (*P* = .2280, *P* = .0951 respectively).

Detailed investigation of the contribution of oligogenicity to genotype-phenotype variability mandates the study of large kindreds with a spectrum of genotypes, eg, F10 (Figure 5). In this large, consanguineous Pakistani kindred, the proband harbors a known pathogenic *DUOX2* mutation (p.Q570L, previously published in ref. 8). Homozygosity for this mutation segregates with permanent CH (F10a), whereas *DUOX2* p.Q570L heterozygotes exhibit either euthyroidism or transient CH. Two novel, rare *TG* variants (p.L2547Q, predicted to be pathogenic by PolyPhen and SIFT, and p.R1691C, of less certain significance) were also identified in this kindred, yet neither of these variants segregated with transient CH in the DUOX2 p.Q570L heterozygotes, suggesting digenic mutations in the genes screened did not explain the phenotypic variability associated with this genotype.

### Unsolved or ambiguous kindreds (Figure 1, Supplemental Table 3)

This group included two cases harboring heterozygous pathogenic *TG* variants; a novel nonsense mutation in F13 (p.Q771*) and a previously described missense mutation in F12 (p.Q870H). An additional case was heterozygous for a frameshift mutation in *TPO* (p.E510Afs*14, F22). Previous reports of CH due to *TG* and *TPO* mutations most commonly involve biallelic mutations; therefore, it is unclear whether the mild or subclinical hypothyroidism was attributable to the monoallelic mutation or whether they harbored a second “hit” not detected by our sequencing methods. Other cases in this category harbored novel heterozygous *TG* missense (p.Y759C, F14) or splice region (c.3433+3_3433+6delGAGT, F15) variants, a novel heterozygous *DUOX2* variant (p.R764W, F25) inherited from a healthy parent and a homozygous *DUOXA2* splice site (c.555–5G>A) variant for which in silico predictions were inconclusive (F27). Nine cases (seven families) remained completely unsolved with no likely disease-causing variants identified. Copy number variant (CNV) analysis was undertaken in individuals who had undergone whole exome sequencing: F13, 15, 33 (ambiguous or unsolved cases) and F3, 6–10 (solved cases); however, no rare CNVs were identified that segregated with disease phenotype in each pedigree.

## Discussion

In this study, NGS technologies enabled efficient screening of eight genes associated with CH and GIS in 49 cases from the United Kingdom, Turkey, Middle East, and Asia, and with a spectrum of biochemical phenotypes. In addition to single-gene mutations, the contribution of oligogenic variants was assessed. Previous genetic evaluations of cohorts of CH with GIS have been less comprehensive, screening fewer genes, or fewer cases with restricted ethnicities (6, 9, 22, 23). The only large-scale multiplex study in CH did not select cases on the basis of thyroid morphology and excluded *TG, SLC26A4,* and *IYD* from its sequencing panel (11). Direct sequencing of *DUOX2, TG, TPO,* and *TSHR* has been undertaken in 43 Korean CH cases with GIS (6); in common with our study, only around 50% of cases harbored causative, pathogenic variants in one or more genes.

The relative frequency of mutations in known CH causative genes depends on selection criteria and ethnic origin of the cohort (6, 24). Our cohort included individuals of diverse ethnicities, in whom the biochemical diagnosis of CH was achieved using different, country-specific, screening protocols, or following neonatal or early childhood presentation with clinical hypothyroidism. These multiple variables preclude detailed comparison of relative mutation frequencies with other studies of populations with more uniform ethnicity or biochemical diagnostic approach. The heterogeneous population screened in this study also mandated the use of ethnically matched controls in order to prevent “false-positive results” due to incorrect classification of ethnically specific single nucleotide polymorphisms as pathogenic mutations. The paucity of West Asian exomes in publically accessible databases precluded this for 17 non-Turkish West Asian cases. However, the large number of controls used (∼80,000) and that eight of the 10 solved West Asian cases harbored truncating or previously reported CH-associated mutations, made false-positive results unlikely.

In our study, mutations were most frequently found in *TG*, followed by *TPO*, whereas *DUOX2* mutations were relatively infrequent compared with findings by Jin et al (mutations in 35% all cases), probably reflecting the higher prevalence of *DUOX2* mutations in individuals of East Asian ethnicity, who were poorly represented in our study (6, 11, 25). No definitively pathogenic mutations were found in *DUOXA2*, *IYD,* or *SLC5A5,* which is in keeping with previous reports suggesting that these are rare genetic causes of dyshormonogenesis, with the exception of a recurrent *DUOXA2* mutation in Korean cases (26, 11). The paucity of *TSHR* mutations in a CH cohort with GIS is surprising; however, the high incidence of consanguinity in our cohort predicts occurrence of biallelic mutations that, in the case of *TSHR*, may cause thyroid hypoplasia, with such cases possibly being excluded from recruitment to our GIS CH cohort (6, 27). Despite unselected recruitment of either sporadic or familial cases, our cohort was greatly enriched for familial CH (76% cases), and consanguinity, which may have increased the percentage of cases harboring an underlying genetic etiology. In a standard United Kingdom clinic population with a greater proportion of sporadic, nonconsanguineous cases, the proportion of mutation-negative cases could be higher.

Interpretation of novel genetic variants requires functional studies in vitro or in vivo evidence of impaired TSH-stimulated mutant thyroglobulin production for *TG* mutations) to confirm pathogenicity (18). Although such analyses were not undertaken, the novel variants identified are rare, segregate with phenotype, and have strong bioinformatic or structural (TPO) predictions of pathogenicity, supporting a causal role. Moreover, the location of novel variants in *TPO* (heme-binding region or substrate-binding region) and *DUOX2* (R354W; peroxidase-like domain) mirrors that of previously described pathogenic mutations. Analysis of novel variants in *TG* is hindered by an incomplete knowledge of its functional domains or crystal structure, but those identified affect similar regions to previously documented mutations (N-terminal cysteine-rich repetitive elements, C-terminal ACHE-like domain) also supporting causality (8, 16, 18, 28).

The associated clinical phenotypes in our mutation-positive patients were similar to published cases. *TG* mutations may result in euthyroid goiter and mild or severe hypothyroidism (18), and monoallelic and biallelic *DUOX2* mutations may both cause permanent or transient CH (8, 21, 23, 25). Even *TPO* mutations, although classically associated with total iodide organification defects, can cause milder phenotypes (28). Solved cases usually had a more severe phenotype than unsolved or ambiguous cases; however, the latter group included four cases of subclinical or mild CH harboring heterozygous mutations in *TPO* or *TG*. Such monoallelic mutations have previously been described in association with CH, but are usually assumed to coexist with an additional undetected CNV, intronic, or regulatory mutation on the other chromosome (16, 24, 29). This may be the case in our patients as well; our sequencing techniques would not have detected mutations in noncoding regions of the genome and, although CNVs were not detected in F15, 13, and 33, they could not be excluded in the remaining families. Our observations highlight that mutations in *TPO* or *TG* may underlie subclinical hypothyroidism as well as cases with overt CH. Despite elevated TSH levels, several of our non-TSHR mutation-positive cases (mainly detected in the neonatal period) did not exhibit goiter. Quantitation of thyroid volume radiologically at this age is technically challenging, such that mild thyroid enlargement may not have been detected. However, TSH-driven goitrogenesis in these cases will have been dependent on fetal TSH levels—whose role in thyroid follicular cell growth remains unclear. In common with our findings, others have demonstrated that dyshormonogenetic CH, even associated with total iodide organification defect, is not always associated with thyroid enlargement (30).

Oligogenicity has often been proposed to underlie the intrafamilial variability seen in known genetic causes of CH, especially in association with *DUOX2* mutations (8). The *Pax8/Titf1* murine model exemplifies the role of polygenicity in thyroid dysgenesis because only mice doubly heterozygous for the two null alleles and bred on a C57BL/6 background exhibit a phenotype (31). Despite reports of digenic GIS cases in the literature, pedigree studies have either not been performed (11, 6) or have not confirmed a genotype-phenotype correlation (12). Our study detected likely pathogenic variants in more than one CH-associated gene, especially in consanguineous kindreds, most commonly involving *TG* and *DUOX2*. It is possible that this is a conservative estimate of the frequency of oligogenicity in CH with GIS; the high percentage of consanguinity in our study facilitates identification of potentially pathogenic variants in a disease model with recessive inheritance, but also increases the likelihood of detecting variants which are contributory to the CH phenotype but not causative, due to the occurrence of genomic regions with loss of heterozygosity involving CH-associated genes. Accordingly, we cannot discount the possibility that some of our monogenic, consanguineous, “solved” cases harbor additional mutations in genes that were not screened in our study, which could contribute to the CH phenotype. Small pedigree sizes, poor information about mutation frequencies in populations matched to our CH cases, and a paucity of subphenotype data preclude definitive statements regarding the relative etiological contribution of digenicity in CH. Further studies with large pedigrees and clear phenotypic variability are required to ascertain the role of polygenic modulators in CH with GIS. Alternative candidate genes involved in the same biological pathways as known causative genes may be implicated, either exacerbating or playing a compensatory role in the context of loss-of-function mutations. Examples include *DUOX1, DUOXA1,* and *NOX*, which are also involved in H_2_O_2_ production and whose expression may be upregulated in the context of *DUOX2* deficiency (12, 32).

It is conceivable that despite adequate median coverage, nonuniform coverage of genes could have resulted in failure to detect variants. This is most likely to be significant for the 11 cases (eight families) in which coverage of specific exons was less than 10-fold (predominantly affecting *DUOXA2* and *SLC5A5*). Suboptimal coverage of these regions raises the possibility of a type II error. However, undetected variants in these cases are unlikely to affect the conclusions of this study because five cases harbored mutations that explained their CH (F26, F2a, b, F11, F17), and two ambiguous cases harbored heterozygous TG variants (F12 a, b). Additionally, although the study was not designed to allow direct comparison of different sequencing methods, the rate of causative mutations in cases screened using either the most sensitive technique (MiSeq targeted sequencing, in which exons with <20-fold coverage were individually resequenced using Sanger sequencing) or WES, was similar and supported our conclusion that approximately 40% cases are unsolved. Previous studies have also reported considerable variability in uniformity and depth of coverage across the exome, and these data, together with our sequencing depth analysis, highlight a limitation of targeted sequencing, which may impact and limit variant identification (33). High-depth, whole-genome sequencing can improve exon coverage and the advent of recent sequencing technologies (such as the Illumina X10 system) makes this possible at large scale.

The etiology of CH with GIS remains elusive, and factors other than known dyshormonogenesis-associated genes or the *TSHR* must be implicated. CH with GIS may be transient, and most of our cases did not undergo a formal trial off levothyroxine withdrawal. However, requirement for ongoing levothyroxine replacement in significant dosage, or continuing TSH elevation, suggested persistent CH in at least 12 unsolved cases. Biochemical CH did tend to be more severe in genetically ascertained cases, which argues against the routine screening of *TG* and *TPO* in milder GIS CH cases. Iodine status was not assessed; however, the high familial component in the unsolved case category favors an etiological contribution of genetic factors rather than environmental modulators, including regulatory region or intronic mutations, or CNVs in the genes screened. Genes associated with syndromic CH (eg, *GLIS3, GNAS*) were not analyzed. Not formally quantitating thyroid gland size might have failed to ascertain cases with mild thyroid hypoplasia, harboring mutations in some thyroid-dysgenesis associated genes (eg, *PAX8, Nkx2–1*). Our aim in using the HiSeq-targeted sequencing and MiSeq protocols was to exclude mutations in known CH-associated genes to identify a smaller, mutation-negative cohort, which could then be analyzed by WES. Thus, future studies with WES/whole genome sequencing in familial cases may identify novel genetic etiologies for CH with GIS, elucidating novel pathways in thyroid development and physiology.

**Note added in proof:** During preparation and revision of this paper, two of the variants which we defined as novel have been described by other groups in association with congenital hypothyroidism: **TG c.638+5G>A** (Li Y, Salfelder A, Schwab KO, *et al*. Against all odds: blended phenotypes of three single-gene defects. *Eur J Hum Genet*. 2016;24:1274–1279) and **DUOX2 c.1060C>T, R354W** (Liu S, Zhang W, Zhang L, *et al*; Genetic and functional analysis of two missense DUOX2 mutations in congenital hypothyroidism and goiter. *Oncotarget*. 2016 doi:10.18632/oncotarget.10525). We would like to acknowledge this work.
